# Case report: Long remission and survival following immunotherapy in a case of pulmonary pleomorphic carcinoma

**DOI:** 10.3389/fimmu.2024.1464900

**Published:** 2024-11-15

**Authors:** Tongshan Wang, Muyang Chen, Anpeng Wang, Hao Zhang

**Affiliations:** ^1^ Central Laboratory, Nanjing First Hospital, Nanjing Medical University, Nanjing, China; ^2^ School of Pediatrics, Nanjing Medical University, Nanjing, Jiangsu, China; ^3^ Department of Oncology, First Affiliated Hospital of Nanjing Medical University, Nanjing, Jiangsu, China

**Keywords:** pleomorphic lung carcinoma, immunotherapy, camrelizumab, apatinib, long remission

## Abstract

In 2020, we reported on a case involving a 68-year-old male patient with a rare instance of pulmonary pleomorphic carcinoma exhibiting high PD-L1 expression. The patient experienced significant therapeutic success with the use of camrelizumab, achieving partial tumor remission. Following the publication of that report, the patient continued on camrelizumab at a dose of 200 mg/dl for 27 cycles, subsequently transitioning to a combination of camrelizumab and bevacizumab for eight cycles. Due to elevated blood pressure, the regimen was adjusted back to monotherapy with camrelizumab. As of July 9, 2024, the patient remains alive with a satisfactory quality of life. This follow-up report, coupled with a review of the literature from 2021 to 2024 on pulmonary pleomorphic carcinoma and its immunotherapeutic approaches, aims to present new insights and innovative strategies for treating this rare form of cancer.

## Introduction

Pulmonary Pleomorphic Carcinoma (PPC) is a rare subtype of non-small cell lung cancer (NSCLC), accounting for 0.4–1.6% of malignant pulmonary tumors. According to the 4th edition of the World Health Organization classification of lung cancers, PPC is defined as a poorly differentiated NSCLC, characterized by the presence of ≥10% spindle or giant cells. These tumors predominantly occur in heavy-smoking males and are known for their poor response to conventional cytotoxic chemotherapy, such as cisplatin, gemcitabine, and paclitaxel, resulting in a worse prognosis compared to other NSCLC types (1). Many PPCs comprise a mix of sarcomatoid (spindle or giant cells or both) and epithelial components (adenocarcinoma, squamous cell carcinoma, or undifferentiated NSCLC) (2, 3). Given the rarity of this tumor type, it can be reasonably inferred that its development is driven by multiple simultaneous genetic mutations and the complex interactions among these genes, which significantly complicates treatment. However, it is noteworthy that >90% of PPC patients exhibit positive PD-L1 expression in tumor tissues, supporting the potential of immunotherapy as a viable option for this patient group (4). This article follows the case of a PPC patient who, after the failure of first-line chemotherapy, achieved partial remission and long-term survival using the PD-1 inhibitor camrelizumab as the primary immunotherapy, a feat that can be described as miraculous.

## Case report

We previously reported this case in 2020 (5). The patient, a 68-year-old male at the time of his first visit in 2019 (now aged 73), is a non-smoker, highlighting the rarity and complexity of this case given that pulmonary pleomorphic carcinoma (PPC) predominantly occurs in heavy-smoking males. At the age of 63, he underwent partial gastrectomy for gastric malignancy. An enhanced CT scan revealed a solitary pulmonary mass measuring 5.1 * 5.8 cm, infiltrating the first and second left ribs of the upper left lobe. Multifocal lymphadenopathy was noted in the mediastinum, bilateral hilar, axillary, abdominal aorta, and mesenteric regions. Brain MRI showed no signs of metastasis. A CT-guided percutaneous lung biopsy of the left lung mass confirmed PPC, clinically staged as T3N3M1c IVB. Extensive lymphadenopathy in the abdominal aorta and mesenteric region may suggest that the lung tumor has metastasized to the gastrointestinal tract, a possibility that has been documented in the existing literature (16). However, at the time, the presence or absence of such metastasis did not impact the overall severity of the patient’s condition or the development of systemic treatment plans; therefore, it was not investigated further. Statistical data indicate that the median survival time for this stage of NSCLC is about 6 months, with only about 10% surviving two years (6). Considering the generally poor prognosis of PPC, the patient’s condition at the initial visit was undoubtedly dire. Genetic testing showed negative mutations in the epidermal growth factor receptor (EGFR) gene, but a PD-L1 tumor proportion score (TPS) > 90% was detected using Burning Rock Dx. After one cycle of cisplatin combined with paclitaxel chemotherapy, the patient experienced significant vomiting and a CT scan diagnosed intestinal obstruction. Symptomatic treatment relieved the symptoms, but a chest CT scan 21 days later showed tumor enlargement to 6.3 * 7.2 cm, indicating poor response to first-line chemotherapy. The patient commenced treatment with camrelizumab, a PD-1 antagonist (200 mg every two weeks) in December 2019. After only two weeks of camrelizumab treatment, the tumor in the upper left lobe significantly reduced, and after two more cycles, five rounds of CT evaluation showed partial remission (PR). During treatment, no severe adverse events were observed except for reactive cutaneous capillary endothelial proliferation (RCCEP), which primarily affected the scalp and trunk. A small molecule vascular endothelial growth factor receptor 2 (VEGFR-2) tyrosine kinase inhibitor was administered to effectively control the severity of RCCEP. Biopsy showed a significant reduction in VEGFR2 expression in tumor tissues. By the time of writing in 2020, camrelizumab monotherapy had been continued for nine cycles, and the tumor diameter was effectively controlled at 2.6 *1.4 cm.

The patient continued camrelizumab monotherapy for another 18 cycles until mid-July 2021. A PET-CT scan on August 31, 2020, showed a left apical lung tumor with increased FDG metabolism and a low-density nodule in the left thyroid gland with mildly increased FDG metabolism. The patient developed multiple hemangiomas during treatment, which significantly improved after treatment with the angiogenesis inhibitor apatinib, considered to be limited-stage tumor bone metastasis. Due to intolerance to apatinib, the drug was discontinued. A nuclear bone imaging CT on March 22, 2021, revealed abnormalities overlapping the posterior ribs and medial edge of the scapula, with bony destruction in the left 1st and 2nd ribs and adjacent vertebral appendages. Multiple assessments indicated stable disease. In late September 2021, the patient began treatment with camrelizumab combined with bevacizumab for eight cycles until early April 2022, during which period a chest and full abdomen enhanced CT evaluation showed stable disease. Due to elevated blood pressure, the treatment reverted to camrelizumab monotherapy in early May 2022, with concurrent bone protective therapy. A chest and abdominal CT on July 26, 2022, indicated stable and manageable tumor condition, with calcification at the liver diaphragm top, multiple small liver cysts, dilated bile ducts, and bilateral renal cysts. Following his discharge in July 2022, the patient has continued maintenance immunotherapy with camrelizumab to date, still alive and with a good quality of life. The **Supplementary Materials** include Chinese medical records that corroborate the relevant diagnostic and treatment processes. The CT report from April reveals that the soft tissue shadow in the apicoposterior segment of the left upper lobe (i.e., the tumor) remains comparable in size to previous imaging, indicating effective tumor control. (The original Chinese transcript and English translation of the CT report will be provided in the **Supplementary Materials**) This suggests possible changes in the tumor’s intrinsic properties (e.g., increased PD-L1 expression, reduction in the proportion of more aggressive sarcomatous components), which may have contributed to its decreased invasiveness. Given the patient’s current improvement, performing further invasive procedures, such as biopsies, would not align with the medical ethical principle that benefits must outweigh risks. Consequently, deeper investigation is not feasible at this stage and requires further studies using animal models.

## Recent research summary:

From 2021 to the present, continuous advancements have been made in the study of pulmonary pleomorphic carcinoma (PPC) and its immunotherapy. This section provides an overview and discussion of these developments.

## In-depth clarification of the heterogeneity in pulmonary pleomorphic carcinoma

PPC is categorized into epithelial and sarcomatoid components, with undeniable phenotypic heterogeneity within and between these components, directly impacting therapeutic outcomes. Masaaki Nagano and colleagues have identified that, within the same PPC tumor samples, the sarcomatoid components tend to express higher levels of PD-L1 compared to the epithelial components, suggesting that immunotherapy with checkpoint inhibitors might be more effective in PPCs with a higher proportion of sarcomatoid components (1). Luca Roma et al. shows that both epithelial and sarcomatoid components of PPC exhibit extensive genomic variations with a common signaling pathway: the RTK-RAS pathway. The significant mutations in this pathway within PPC cells suggest its crucial role in PPC evolution and its potential as a novel therapeutic target in PPC treatment. Additionally, the epithelial-to-mesenchymal transition (EMT), marked by increased NCAM1 and decreased CDH1 expression, controls the transformation between clonally related tumor components from epithelial to sarcomatoid-like states (7). This process could potentially be targeted to convert more epithelial cells into sarcomatoid cells, enhancing the cytotoxic effect of immune checkpoint inhibitors on PPC. Supporting Chinese case records that corroborate the aforementioned diagnostic and treatment processes can be found in the **Supplementary Materials**. The CT report from April this year (**Supplementary Materials**) indicates that the soft tissue mass (tumor) in the apicoposterior segment of the left upper lobe has a similar extent as before, suggesting effective control of the tumor’s spread. There is also a likelihood that the intrinsic characteristics of the tumor have undergone changes, such as an increase in PD-L1 content and a reduction in the proportion of more aggressive sarcomatous components due to targeted killing, thereby reducing its aggressiveness. However, given the patient’s improvement, further biopsy studies would not align with the medical ethical principle of ‘benefit outweighing harm,’ and thus, deeper investigation is not feasible. Further research based on animal models is warranted to explore this further.

## Prognostic models for pulmonary pleomorphic carcinoma

A comprehensive risk model based on semantic and radiomic features has been developed by Chohee Kim’s team, providing superior prognostic predictions compared to using semantic or radiomic features alone. Their findings suggest that a high SUVmax in the solid portion of the tumor (CT first-order energy) is associated with poor prognosis in pulmonary PC (8), possibly due to the rapid metabolism and proliferation of tumor tissues. Hideko Onoda’s team points out that the presence of air bronchograms in imaging studies typically indicates a relatively good prognosis for PPC patients, as this sign often denotes lower aggressiveness of the tumor (10).

## Latest advances in immunotherapy and similar cases for pulmonary pleomorphic carcinoma

Following our case report published in 2021, Japanese scholars reported three cases of PPC treated with immune checkpoint inhibitors. A 73-year-old male, similar to the patient discussed in our report, received pembrolizumab monotherapy—more commonly used internationally—but showed no response and passed away after four months. Another 66-year-old male who was treated with a combination of pembrolizumab, carboplatin, and albumin-bound paclitaxel showed significant improvement, suggesting that immune checkpoint inhibitors might enhance the sensitivity of PPC to chemotherapy. Additionally, a 49-year-old male treated with pembrolizumab monotherapy for second-line treatment of PPC demonstrated tumor size reduction after 11 months in imaging studies (9), indicating that immune checkpoint inhibitors remain the best option for PPC treatment until breakthroughs in targeted therapies are achieved.

## Discussion

Pulmonary pleomorphic carcinoma (PPC) is a rare malignant tumor characterized by a dual-cell component of spindle and/or giant cells alongside epithelial cells. Accordingly, effective treatment options are extremely limited.For patients diagnosed with PPC who present without lymph node involvement and distant metastasis (N0M0), the standard treatment approach typically involves surgical resection. Conversely, in cases where lymph node involvement or distant metastasis is detected, palliative chemotherapy is usually employed, utilizing regimens similar to those administered to other NSCLC patients. These regimens may include platinum-based drugs, paclitaxel, docetaxel, and others. However, it is important to note that the efficacy of these palliative treatments in this context is often significantly limited (17–19). According to a meta-analysis, even with early surgical resection (pN0) followed by standard adjuvant therapy, the 5-year survival rate remains below 40%. Once recurrence occurs, the median survival duration drops to just 2.6 months (11). A key feature of PPC is the exceptionally high PD-L1 positivity rate, particularly within the sarcomatoid components (1, 4), the reasons for which are not yet clear. It is hypothesized that this may be related to hypoxic conditions and inflammatory responses. Existing studies have shown that high PD-L1 levels in PPC are also associated with lymph node metastasis and pleural invasion, often indicating a poor prognosis (12), which aligns with the case we report here, where the tumor initially invaded the mediastinal pleura with extensive lymph node metastasis.

Camrelizumab, a programmed cell death 1 (PD-1) inhibitor developed by Jiangsu Hengrui Pharmaceuticals Co., Ltd., has recently been conditionally approved in China for the treatment of relapsed or refractory classical Hodgkin lymphoma. This drug is also being investigated for various other malignancies, including B-cell lymphoma, esophageal squamous cell carcinoma, gastroesophageal junction cancer, hepatocellular carcinoma, nasopharyngeal carcinoma, and non-squamous, non-small cell lung cancer (13). Compared to the internationally used PD-1 inhibitors such as pembrolizumab by Merck and nivolumab by BMS, camrelizumab offers a lower cost and better cost-effectiveness, though it has limited recognition. Reactive cutaneous capillary endothelial proliferation is one of the most common complications of camrelizumab, with an incidence rate as high as 88%. In addition to the use of apatinib to counteract angiogenesis in this case, another therapeutic approach—5-aminolevulinic acid photodynamic therapy (ALA-PDT)—has been reported. It successfully alleviated multiple skin lesions on the chest and buttocks of patients within a week, with no recurrence after six months (14). There is currently a Phase I study on the combination of camrelizumab and apatinib in advanced gastric cancer, showing promising results with a confirmed objective response rate (ORR) of 76.5% (95% confidence interval [CI] 58.8–89.3) and a disease control rate (DCR) of 91.2% (76.3–98.1), extending the median duration of response (DOR) by 7.6 months (5.4–not assessable) (15). In another clinical study using these two drugs in combination for second-line treatment of advanced squamous non-small cell lung cancer, the median follow-up time was 13.3 months (range: 1.6 to 19.2 months). Among all 25 participants, the ORR was 32.0% (95% CI: 14.9% to 53.5%), the clinical benefit rate was 44.0% (95% CI: 24.4% to 65.1%), and the disease control rate (DCR) was 84.0% (95% CI: 63.9% to 95.5%). The median progression-free survival (PFS) was 6.0 (95% CI: 3.5 to 8.1) months, and the median overall survival (OS) was 13.3 (95% CI: 6.4 to 18.8) months. The clinical benefit of this combination was evident in all tumor PD-L1 expression subgroups, suggesting that it has good anti-tumor activity against general non-central squamous NSCLC (20). Given the high PD-L1 expression, strong invasive and metastatic capabilities, and vascular richness of PPC, there is potential for applying this drug combination in the treatment of PPC in the future. In addition, monoclonal antibodies that specifically target PD-L1, such as atezolizumab, represent potential treatment options for pulmonary pleomorphic carcinoma and have been utilized in clinical practice. However, it is noteworthy that the patient with pulmonary pleomorphic carcinoma in this case ultimately succumbed to respiratory failure just 13 days after the administration of atezolizumab (21). Overall, due to the rarity of this cancer, initiating large-scale clinical trials may be challenging, and treatment methods must rely on clinicians’ integration of past literature and personal experience, involving considerable uncertainty.

## Conclusion

Through the case report of a rare instance of pulmonary pleomorphic carcinoma (PPC) achieving remission and prolonged survival with camrelizumab, along with a review of selected literature, we conclude that camrelizumab, as a primary agent supplemented by anti-angiogenic drugs such as apatinib and conventional chemotherapy, holds promise as a significant therapeutic option for patients with PPC in the future. Unfortunately, due to the rarity of pleomorphic lung cancer, most of the literature available, including the present study, consists primarily of case analyses. As a result, we have been unable to locate large-scale statistical analyses or comprehensive clinical trial data on this condition. Although we have made every effort to summarize the general conclusions based on these individual cases and the available data, there are inherent limitations regarding the persuasiveness of our findings. These limitations must be addressed through further research conducted by other investigators in the field.

## Data Availability

The original contributions presented in the study are included in the article/**Supplementary Material**. Further inquiries can be directed to the corresponding author/s.
