# Corrigendum: Case report: Long remission and survival following immunotherapy in a case of pulmonary pleomorphic carcinoma

**DOI:** 10.3389/fimmu.2024.1530843

**Published:** 2024-12-03

**Authors:** Tongshan Wang, Muyang Chen, Anpeng Wang, Hao Zhang

**Affiliations:** ^1^ Central Laboratory, Nanjing First Hospital, Nanjing Medical University, Nanjing, ;China; ^2^ School of Pediatrics, Nanjing Medical University, Nanjing, Jiangsu, ;China; ^3^ Department of Oncology, First Affiliated Hospital of Nanjing Medical University, Nanjing, Jiangsu, ;China

**Keywords:** pleomorphic lung carcinoma, immunotherapy, camrelizumab, apatinib, long remission

In the published article, there was an error. Camrelizumab was erroneously referred to as pembrolizumab.

A correction has been made to the **Abstract**. This sentence previously stated:

“In 2020, we reported on a case involving a 68-year-old male patient with a rare instance of pulmonary pleomorphic carcinoma exhibiting high PD-L1 expression. The patient experienced significant therapeutic success with the use of pembrolizumab, achieving partial tumor remission. Following the publication of that report, the patient continued on pembrolizumab at a dose of 200 mg/dl for 27 cycles, subsequently transitioning to a combination of pembrolizumab and bevacizumab for eight cycles. Due to elevated blood pressure, the regimen was adjusted back to monotherapy with pembrolizumab. As of July 9, 2024, the patient remains alive with a satisfactory quality of life. This follow-up report, coupled with a review of the literature from 2021 to 2024 on pulmonary pleomorphic carcinoma and its immunotherapeutic approaches, aims to present new insights and innovative strategies for treating this rare form of cancer.”

The corrected sentence appears below:

“In 2020, we reported on a case involving a 68-year-old male patient with a rare instance of pulmonary pleomorphic carcinoma exhibiting high PD-L1 expression. The patient experienced significant therapeutic success with the use of camrelizumab, achieving partial tumor remission. Following the publication of that report, the patient continued on camrelizumab at a dose of 200 mg/dl for 27 cycles, subsequently transitioning to a combination of camrelizumab and bevacizumab for eight cycles. Due to elevated blood pressure, the regimen was adjusted back to monotherapy with camrelizumab. As of July 9, 2024, the patient remains alive with a satisfactory quality of life. This follow-up report, coupled with a review of the literature from 2021 to 2024 on pulmonary pleomorphic carcinoma and its immunotherapeutic approaches, aims to present new insights and innovative strategies for treating this rare form of cancer.”

Additionally, due the same error, several corrections have been made to the main text. A correction has been made to **Case report** Paragraph 1. This sentence previously stated:

“The patient commenced treatment with pembrolizumab, a PD-1 antagonist (200mg every two weeks) in December 2019. After only two weeks of pembrolizumab treatment, the tumor in the upper left lobe significantly reduced, and after two more cycles, five rounds of CT evaluation showed partial remission (PR). During treatment, no severe adverse events were observed except for reactive cutaneous capillary endothelial proliferation (RCCEP), which primarily affected the scalp and trunk. A small molecule vascular endothelial growth factor receptor 2 (VEGFR-2) tyrosine kinase inhibitor was administered to effectively control the severity of RCCEP. Biopsy showed a significant reduction in VEGFR2 expression in tumor tissues. By the time of writing in 2020, pembrolizumab monotherapy had been continued for nine cycles, and the tumor diameter was effectively controlled at 2.6 *1.4 cm.”

The corrected sentence appears below:

“The patient commenced treatment with camrelizumab, a PD-1 antagonist (200 mg every two weeks) in December 2019. After only two weeks of camrelizumab treatment, the tumor in the upper left lobe significantly reduced, and after two more cycles, five rounds of CT evaluation showed partial remission (PR). During treatment, no severe adverse events were observed except for reactive cutaneous capillary endothelial proliferation (RCCEP), which primarily affected the scalp and trunk. A small molecule vascular endothelial growth factor receptor 2 (VEGFR-2) tyrosine kinase inhibitor was administered to effectively control the severity of RCCEP. Biopsy showed a significant reduction in VEGFR2 expression in tumor tissues. By the time of writing in 2020, camrelizumab monotherapy had been continued for nine cycles, and the tumor diameter was effectively controlled at 2.6 *1.4 cm.”

A correction has also been made to **Case report**, Paragraph 2. This sentence previously stated:

“The patient continued pembrolizumab monotherapy for another 18 cycles until mid-July 2021.”

The corrected sentence appears below:

“The patient continued camrelizumab monotherapy for another 18 cycles until mid-July 2021.”

The correction was also applied later in the same section and paragraph. This sentence previously stated:

“In late September 2021, the patient began treatment with pembrolizumab combined with bevacizumab for eight cycles until early April 2022, during which period a chest and full abdomen enhanced CT evaluation showed stable disease. Due to elevated blood pressure, the treatment reverted to pembrolizumab monotherapy in early May 2022, with concurrent bone protective therapy. A chest and abdominal CT on July 26, 2022, indicated stable and manageable tumor condition, with calcification at the liver diaphragm top, multiple small liver cysts, dilated bile ducts, and bilateral renal cysts. Following his discharge in July 2022, the patient has continued maintenance immunotherapy with pembrolizumab to date, still alive and with a good quality of life.”

The corrected sentence appears below:

“In late September 2021, the patient began treatment with camrelizumab combined with bevacizumab for eight cycles until early April 2022, during which period a chest and full abdomen enhanced CT evaluation showed stable disease. Due to elevated blood pressure, the treatment reverted to camrelizumab monotherapy in early May 2022, with concurrent bone protective therapy. A chest and abdominal CT on July 26, 2022, indicated stable and manageable tumor condition, with calcification at the liver diaphragm top, multiple small liver cysts, dilated bile ducts, and bilateral renal cysts. Following his discharge in July 2022, the patient has continued maintenance immunotherapy with camrelizumab to date, still alive and with a good quality of life.”

