# Supplementary material for: Case report: Long remission and survival following immunotherapy in a case of pulmonary pleomorphic carcinoma
Source: Front Immunol. 2024 Nov 15;15:1464900. doi: 10.3389/fimmu.2024.1464900 (PMC11604722; doi:10.3389/fimmu.2024.1464900)

Medical Record (Final Recorded Entry on 2023.12.07):

Initial Presentation:

October 2019: The patient experienced left back numbness, tingling, and pain, which gradually worsened. On October 29, 2019, a CT scan at a local hospital revealed a soft tissue mass in the left lung apex, approximately 5-6 cm in diameter with irregular margins and disrupted local architecture. Cortical blurring and discontinuity with patchy low-density bone destruction were observed in the left 1st-2nd posterior ribs. No significant lymphadenopathy was noted in the mediastinum or hila. The diagnosis was left upper lung apex cancer with invasion of the left 1st-2nd ribs.

Subsequent Diagnostic Workup and Treatment:

October 30, 2019: Referred to our hospital for further evaluation and biopsy.

November 5, 2019: CT scan confirmed left upper lobe apical posterior mass, suggestive of lung cancer with mediastinal, bilateral hilar, axillary, para-aortic, and mesenteric lymphadenopathy indicative of metastasis. Multiple solid nodules in the left upper and lower lobes suggested pulmonary metastasis. Nodular thickening in the left adrenal gland was also noted.

November 5, 2019: Bone ECT showed no significant abnormalities.

November 8, 2019: MRI of the brain revealed minor lacunar infarctions in the right frontal lobe.

November 5, 2019: Lung biopsy pathology from the left lower lung revealed malignancy, likely invasive carcinoma. Immunohistochemistry indicated CK-pan(+), CK5/6(-), CK-L(+), P40(-), p63(positive at the lesion), TTF-1(-), NapsinA(-), Syn(-), CgA(-), Ki67(>75%). HE staining and immunophenotyping suggested malignancy with epithelial differentiation, lacking clear evidence of pulmonary origin. Following further clinical and imaging assessments, the diagnosis was confirmed as pulmonary pleomorphic carcinoma.

November 12, 2019: Placement of an infusion port.

November 13, 2019: Initiated chemotherapy with liposomal paclitaxel (210 mg, day 1) and cisplatin (40 mg, days 1-3).

Genetic Testing and Follow-up:

November 25, 2019: CT scan during emergency visit due to significant nausea and poor appetite post-chemotherapy, showed progression of the lung tumor, stable abdominal lymphadenopathy, and slight pancreatic duct dilation. Emergency symptomatic treatment was provided.

November 2019 - July 2021: The patient received 27 cycles of immunotherapy with camrelizumab (200 mg, day 1), resulting in initial tumor shrinkage followed by stabilization. PET-CT on August 31, 2020, showed increased FDG uptake in the left lung apex, consistent with malignancy, and a low-density nodule in the left thyroid gland.

September 27, 2021: Commenced treatment with camrelizumab (200 mg) combined with bevacizumab (300 mg) for 8 cycles until April 7, 2022. Discontinued bevacizumab due to elevated blood pressure.

May 3, 2022 - June 29, 2022: Continued with camrelizumab monotherapy (200 mg) for three cycles, accompanied by bone protection therapy.

July 26, 2022: Chest and abdominal CT showed stable soft tissue mass in the left upper lobe and solid nodules in the left lower lobe, with mediastinal, para-aortic, and mesenteric lymph nodes appearing largely unchanged.

Subsequent Treatments: Continued regular camrelizumab (200 mg) therapy in outpatient settings, with follow-up imaging indicating stable disease.

Most Recent Evaluations:

September 13, 2023: Color Doppler ultrasound showed a hypoechoic solid lesion in the left thyroid (10x7x5 mm).

October 30, 2023: Multi-detector CT revealed stable left upper lobe soft tissue mass and solid nodule in the left lower lobe, with stable lymph nodes and multiple small liver cysts compared to April 14, 2023. Slight progression in bile duct and pancreatic duct dilation was noted.

Clinical Status:

Recent Assessments: No significant changes in the patient's condition, no fever, chills, nausea, vomiting, abdominal pain, or diarrhea. Mental status, appetite, sleep, bowel movements, and weight remain stable.

The original Chinese medical record with the patient's name and address hidden is attached：


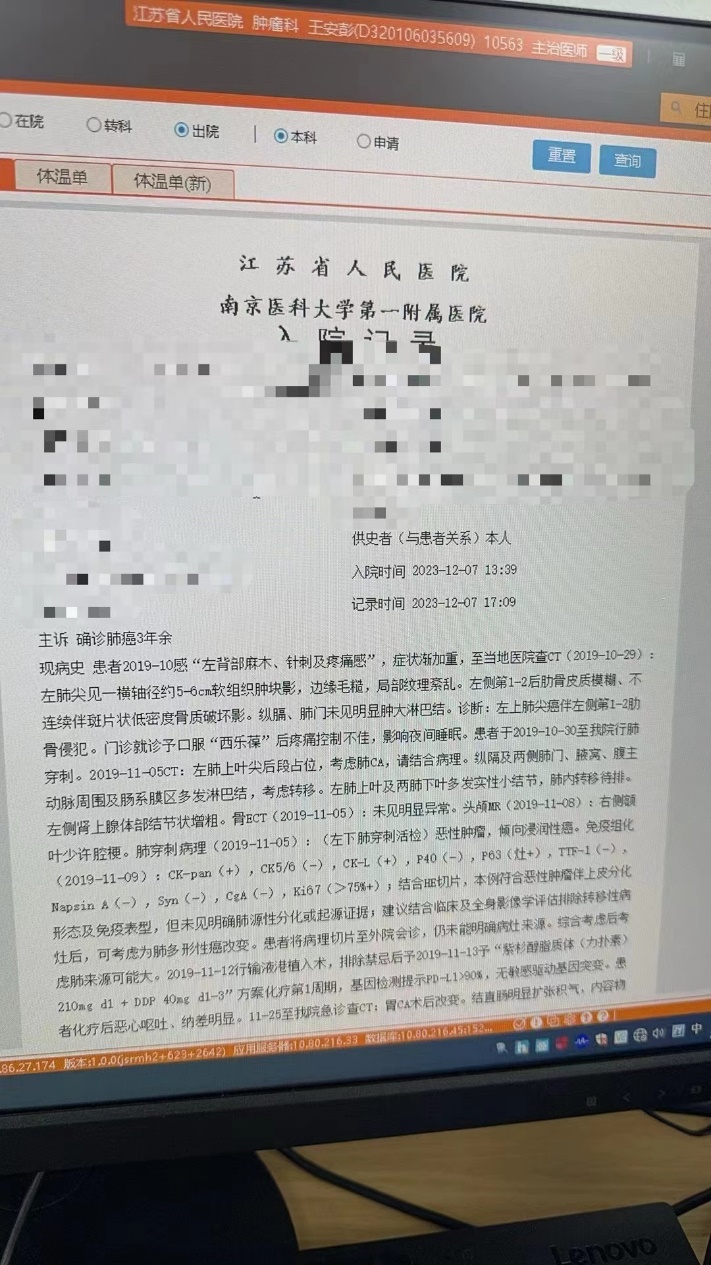

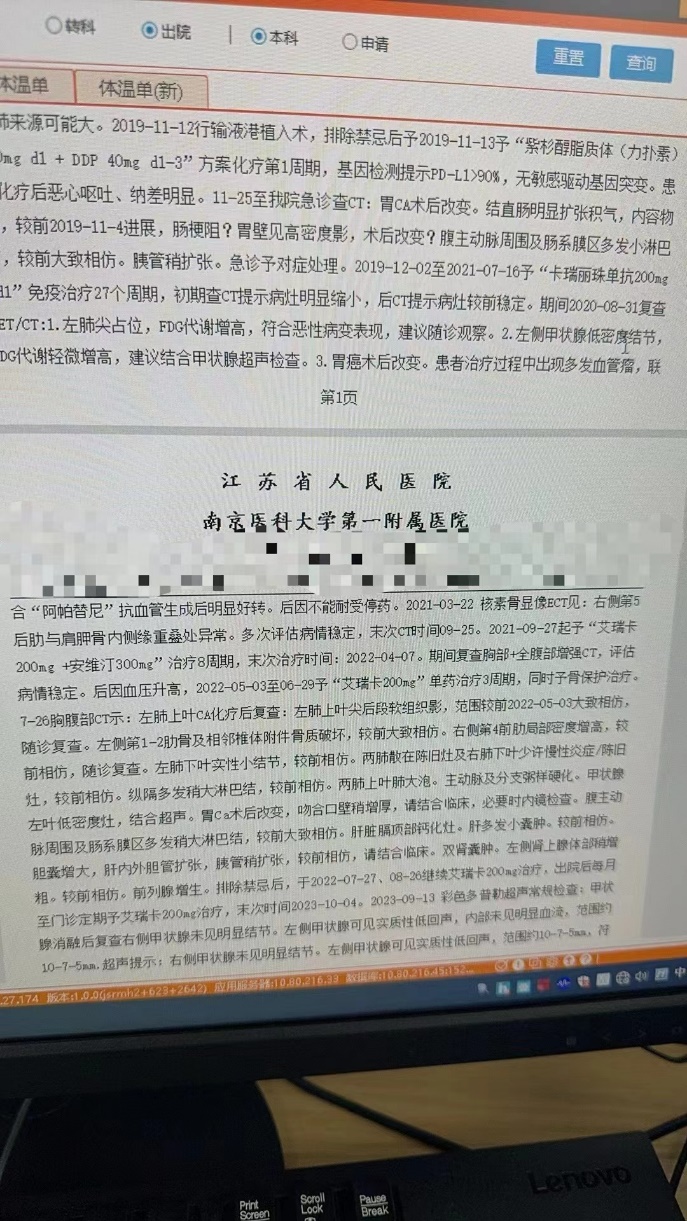


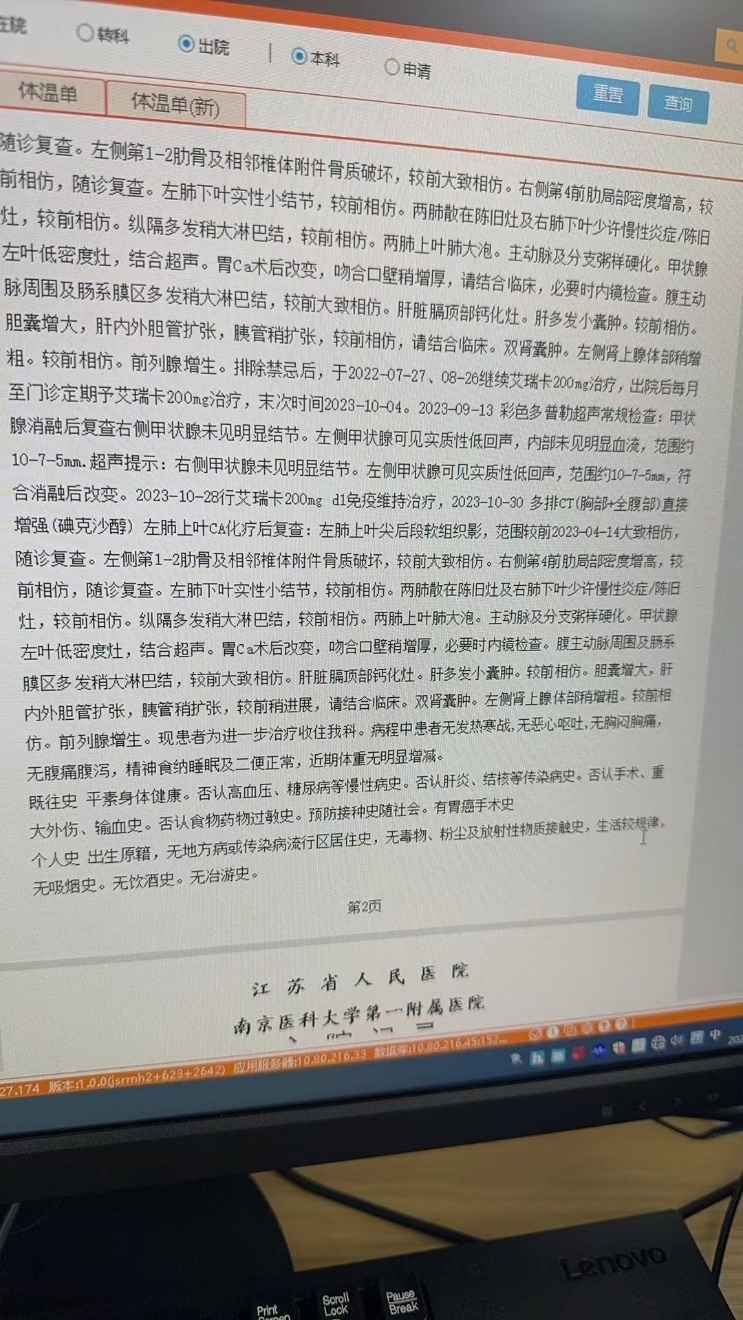

Supplement: Supplementary file 1 [file Table1.docx]
