# Supplementary material for: Case report: Long remission and survival following immunotherapy in a case of pulmonary pleomorphic carcinoma
Source: Front Immunol. 2024 Nov 15;15:1464900. doi: 10.3389/fimmu.2024.1464900 (PMC11604722; doi:10.3389/fimmu.2024.1464900)
Supplement: Supplementary file 2 [file Table2.docx]

CT records:

CT Report (April 19, 2024)

Findings:

Left Upper Lung: There is an irregular patchy area of soft tissue density in the posterior segment of the left upper lobe. The margins are irregular and the internal density is heterogeneous. Surrounding areas show patchy, indistinct opacities.

Left Upper Lobe: There is thickening of the interlobular septa.

Left Lower Lobe: There is a solid nodule with a diameter of less than 5mm.

Both Lungs: Scattered reticular opacities and fibrous streaks are noted.

Right Lower Lobe: There is a patchy, cotton-wool-like opacity adjacent to the spine.

Both Upper Lobes: Multiple subpleural cystic lucencies are observed.

Mediastinum: Multiple slightly enlarged lymph nodes are noted, with the largest having a short diameter of 9mm.

Heart: Normal size.

Aorta and Branches: Atherosclerosis is present.

Bones: Partial absorption and destruction of the bone in the left 1st and 2nd ribs and adjacent vertebral accessory parts. Localized increased density in the right 4th rib.

Thyroid: No enhancement in the low-density area in the left lobe, with a diameter of approximately 1.2cm.

Conclusion:

The tumor has been effectively controlled.

**Original Chinese report and CT images can be found in the attached files.**

**
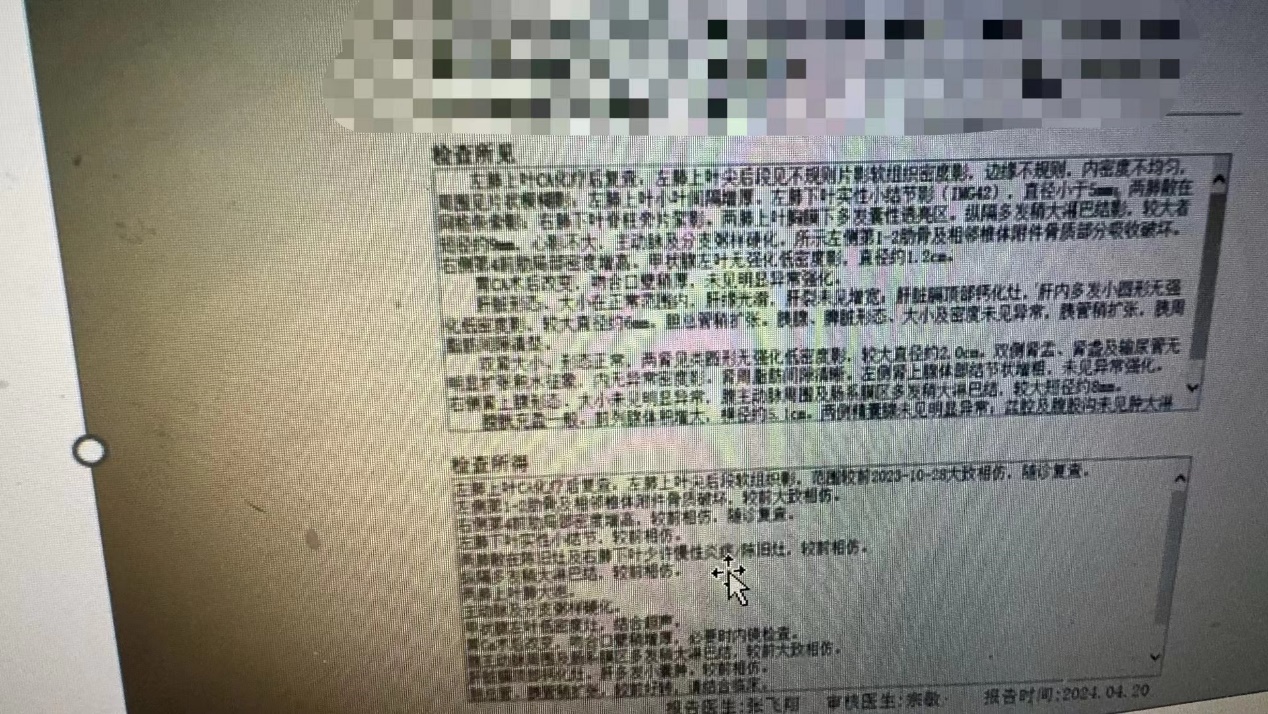
**

**
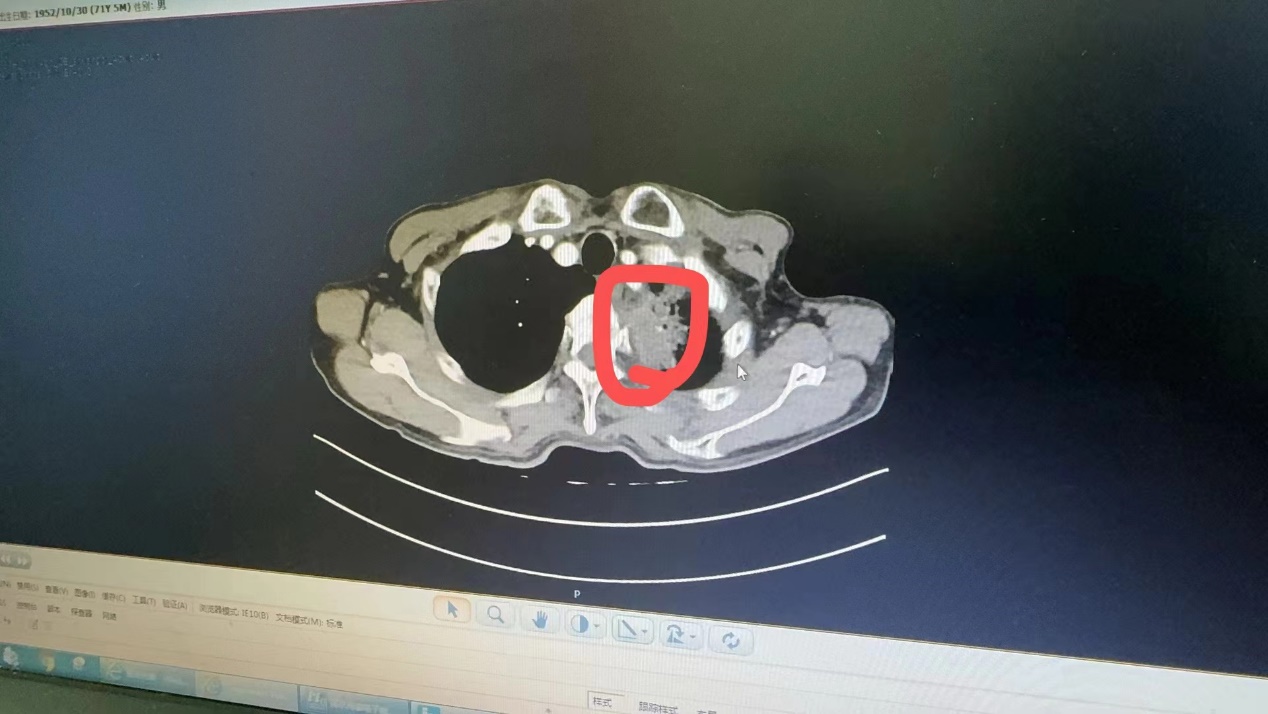
**
